# Disruption of the open conductance in the β-tongue mutants of Cytolysin A

**DOI:** 10.1038/s41598-018-22009-1

**Published:** 2018-02-28

**Authors:** Monifa A. Fahie, Lucas Liang, Alzira R. Avelino, Bach Pham, Patanachai Limpikirati, Richard W. Vachet, Min Chen

**Affiliations:** 1 Molecular and Cellular Biology Program, University of Massachusetts Amherst, Amherst, MA 01003 USA; 2 Department of Chemistry, University of Massachusetts Amherst, Amherst, MA 01003 USA

## Abstract

Cytolysin A (ClyA) is a water-soluble alpha pore-forming toxin that assembles to form an oligomeric pore on host cell membranes. The ClyA monomer possesses an α-helical bundle with a β-sheet subdomain (the β-tongue) previously believed to be critical for pore assembly and/or insertion. Oligomerization of ClyA pores transforms the β-tongue into a helix-turn-helix that embeds into the lipid bilayer. Here, we show that mutations of the β-tongue did not prevent oligomerization or transmembrane insertion. Instead, β-tongue substitution mutants yielded pores with decreased conductance while a deletion mutation resulted in pores that rapidly closed following membrane association. Our results suggest that the β-tongue may play an essential structural role in stabilizing the open conformation of the transmembrane domain.

## Introduction

Pore forming toxins (PFTs) contribute significantly to virulence in many important pathogenic bacteria^1^. PFT permeabilize host cells by creating water-filled channels in their membranes, which leads to cell lysis^2,3^. The alpha (α) and beta (β) classification of PFTs refers to the secondary structure of their transmembrane domain – either α-helices or β -barrels^4^. PFTs are typically secreted as soluble monomeric proteins that bind host membranes in a lipid-dependent manner^5,6^ or through specific protein-receptor interactions^7–9^. Membrane-associated monomers then assemble to form a homo or hetero-oligomeric pre-pore. The pre-pore then converts into a lytic pore by forming the transmembrane domain which crosses the lipid bilayer^2,10^.

**Figure 1:**
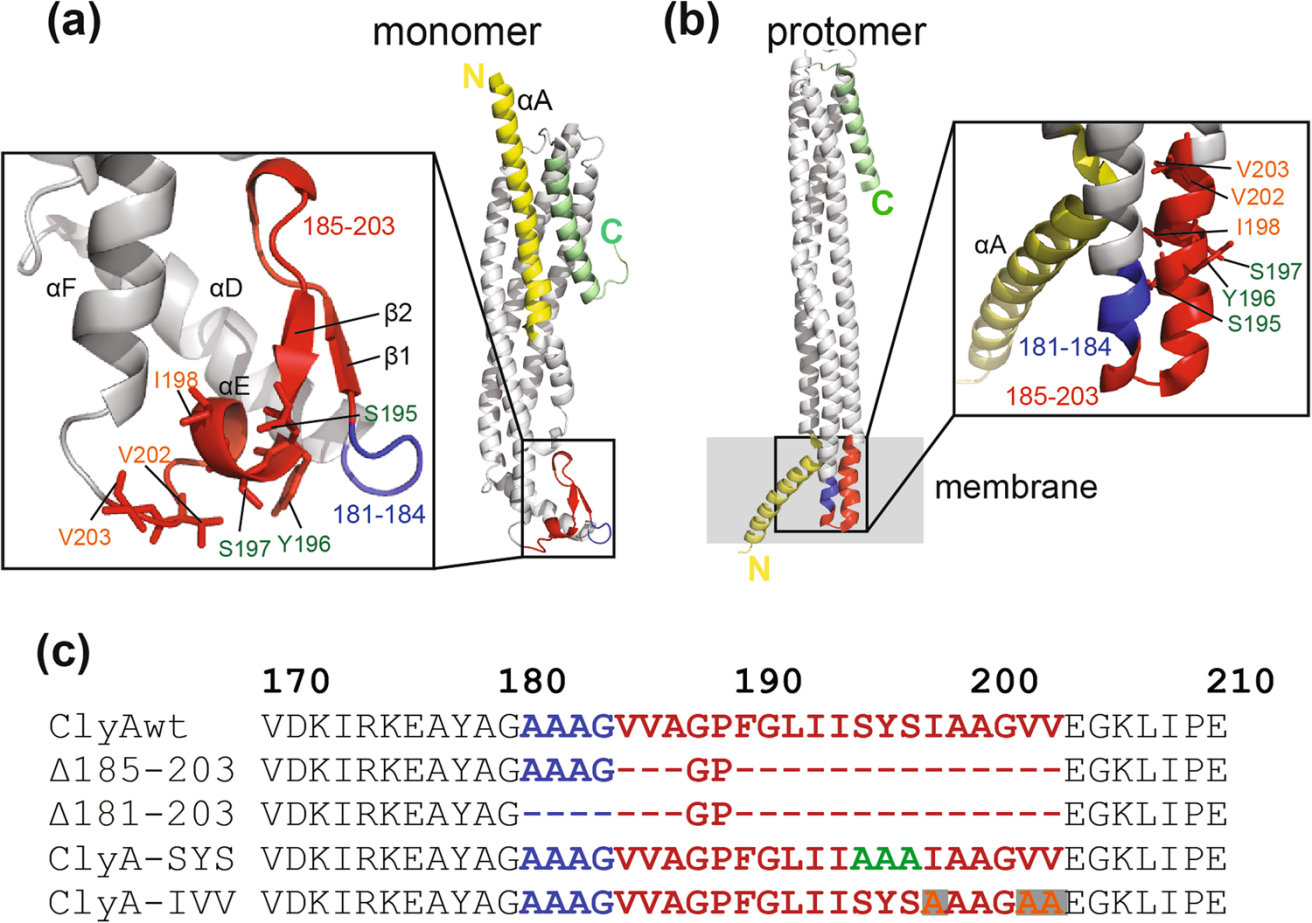
Structure and sequence of ClyA and the β-tongue mutants. Structures of (**a**) the ClyA monomer (1QOY) and (**b**) a protomer from the transmembrane oligomer (2WCD). (**c**) Sequence alignment of ClyA proteins. Mutants Δ185-203 and Δ181-203 have deleted residues indicated by the dashes (−). ClyA-SYS has alanine mutations at S195, Y196, S197 while ClyA-IVV has alanine mutations at I198, V202 and V203.

Cytolysin A (ClyA) is an α-PFT^11,12^ and a virulence factor for several bacterial species such as *Escherichia coli* (*E. coli*)^13–15^ and *Salmonella typhi (S. typhi)*^16^. Unlike classic PFTs, ClyA is secreted into the extracellular environment in outer membrane vesicles (OMVs)^17,18^. Little is known about how the OMVs deliver the cargo toxins to carry out the cytotoxic function^19^. The monomer of ClyA has a rod-shaped helical bundle of four long helices^11^. At one end, the C-terminus of ClyA forms a shorter helix that packs against the αA and αB helices forming a five-helix bundle for about one-third of the length of the monomer (Fig. 1a)^11^. Between the third and fourth helices of the main bundle, a highly hydrophobic β-hairpin (called the β-tongue) was predicted to be the transmembrane region by several groups^11,20^. Indeed, substitution of the hydrophobic residues within the β-tongue with aspartate or serine abolished its cytolytic activity, thus suggesting its role in forming the transmembrane domain^21–23^. However, the crystal structure of the ClyA transmembrane pore revealed that a large conformational change occurring during the conversion of the soluble monomer to the dodecameric transmembrane pore (Fig. 1b): the N-terminal amphipathic αA helix rotates 180° to pack with αA helices of neighboring monomers to form an iris-like α-helical barrel^24^. This conformational change is critical for protomer assembly as fixing the N-termini to the C-terminal helix using an engineered disulfide prevented the oligomerization^25^. Surprisingly, the β-tongue was not a component of the transmembrane barrel. Instead, it converted into an alpha helix-turn-helix that was located adjacent to the transmembrane domain for roughly 30% of the barrel length^24^. Therefore it was proposed that the β-tongue may act as a hydrophobic membrane anchor that initiates the membrane association which subsequently triggers the assembly of the oligomer^24^. However, earlier biochemical studies do not fully support this notion as deletion or substitution mutations in the β-tongue region did not completely abolish the membrane association ability^21,22^.

To understand why the β-tongue is necessary for the cytolytic function of ClyA, we created two β-tongue deletion mutants, two substitution mutants and explored their ability for: (1) membrane association; (2) oligomerization and (3) pore formation. Our data showed that the mutants retained the ability to interact with the membrane and form ring-like oligomers that were comparable to the wild type. However, the hemolytic activity of these mutants was either severely impaired or abolished, which correlated with the reduction or loss of the open conductance in the lipid bilayer. Our results suggest that the β-tongue domain may be involved in maintaining the open conformation of the α-helical transmembrane barrel.

## Results

### Characterization of the β-tongue mutants

**Figure 2:**
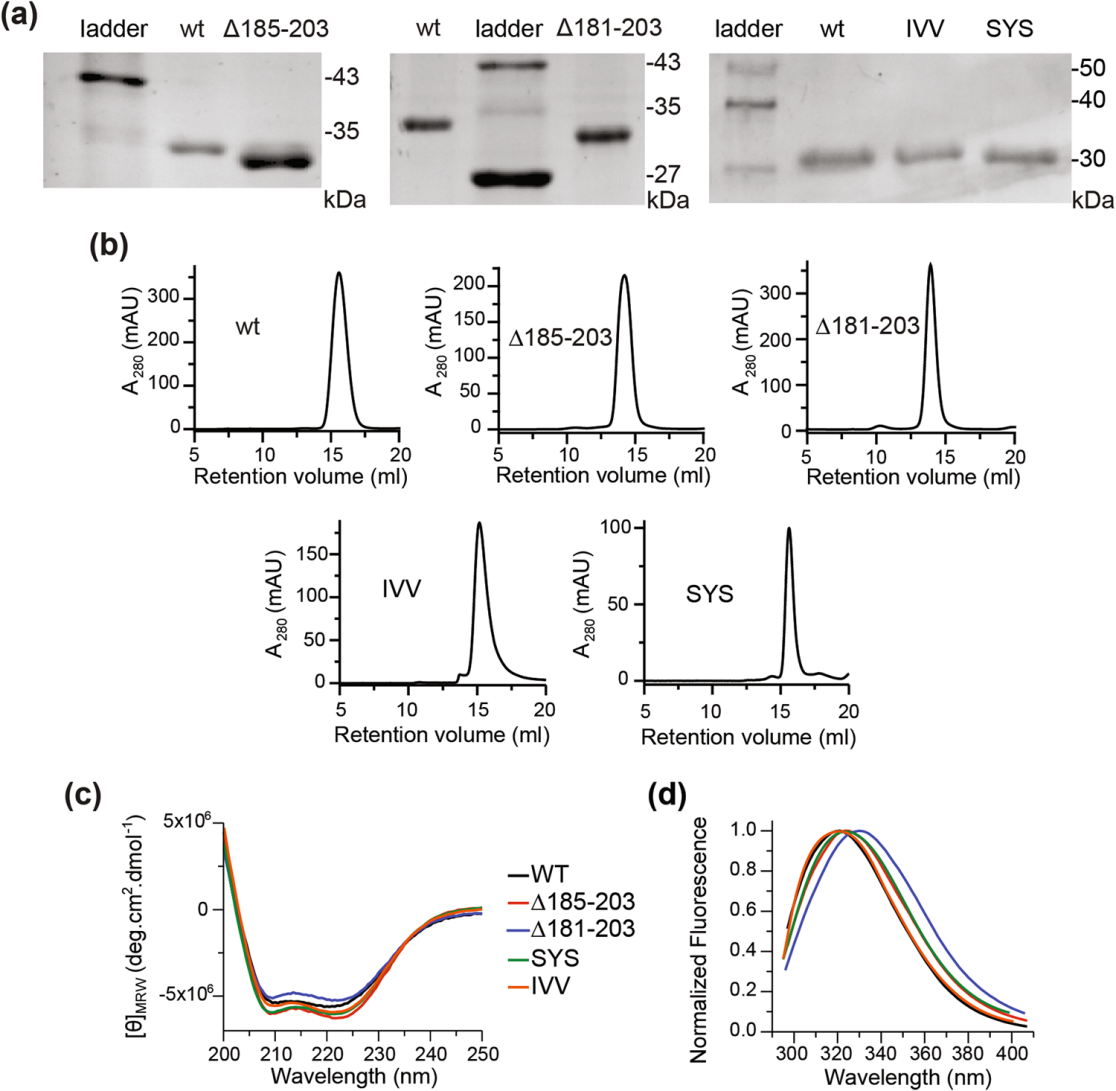
Characterization of ClyA proteins. (**a**) SDS-PAGE of purified WT protein, Δ185–203, Δ181–203, IVV and SYS mutants on separate gels which were cropped. (**b**) Gel filtration profiles of the purified proteins using Superdex 200 column (GE). (**c**) Secondary structure analysis of ClyA samples using circular dichroism. (**d**) Intrinsic fluorescence spectra of ClyA samples excited at 280 nm and fluorescence read from 293–407 nm.

We created two deletion mutants and two substitution mutants to test how the β-tongue region of ClyA affects its assembly and pore-forming ability. One mutant had a deletion of residues 185–203, corresponding to the two β strands and the helix αE only, and a slightly longer deletion where residues 181–203 were removed (Fig. 1). The glycine 188 and proline 189 were kept in the truncated region (Fig. 1c) to form the turn connecting the end of two helices that run parallel to each other in the protomer structure (Fig. 1a). The two other mutants were created to test how altering the hydrophobicity in the β-tongue would affect ClyA. The mutant with decreased hydrophobicity was I198A/V202A/V203A (ClyA-IVV) while the mutant with increased hydrophobicity was S195A/Y196A/S197A (ClyA-SYS). As expected, the purified Δ185–203 mutant and Δ181–203 mutant ran faster than ClyA wild-type (ClyAwt) in SDS-PAGE (Fig. 2a). The average molecular mass of the ClyA proteins were confirmed by mass spectrometry to be 34.447 kDa for ClyAwt, 32.790 kDa for Δ185–203, 32.650 kDa for Δ181–203, 34.330 kDa for ClyA-SYS and 34.350 kDa for ClyA-IVV which were all within 3 Da of their theoretical mass (Supplementary Figure S1).

Interestingly, the deletion mutants in their native state eluted earlier than ClyAwt in gel filtration suggesting a partially misfolded or altered monomeric state (Fig. 2b). Dynamic light scattering measurements however suggest that the Δ185–203 mutant was dimeric, while the Δ181–203 mutant was trimeric (Supplementary Table S1). It is unclear why the deletion of the β-tongue could lead to oligomer formation but a possible explanation is that the deleted region of the mutants may have disrupted the packing of the α-helix bundle resulting in the partial exposure of hydrophobic regions that promoted the non-native oligomerization of monomers.

To assess whether the secondary structures were affected, ClyAwt and the mutants were characterized using circular dichroism (Fig. 2c). Both deletion and substitution mutants showed significant alpha helical content, indicating the secondary structures were largely retained. In addition, we obtained the intrinsic fluorescence of the proteins (Fig. 2d). ClyA has two tryptophan residues, W37 and W86 which are both buried in the monomeric form (Supplementary Figure S2). The intrinsic fluorescence of ClyA-IVV mutant aligned well with that of ClyAwt suggesting a very similar fold. However, the emission maximum for ClyA-SYS and Δ185–203 mutants was red shifted by 4 nm while there was a 7 nm red shift for the Δ181–203 mutant (Fig. 2d, Supplementary Figure S3). Notably, all mutants had a reduced fluorescence intensity compared to ClyAwt (Supplementary Figure S3). The red shift in emission maximum as well as the reduction in fluorescence intensity suggests that either one or both of the tryptophan residues of the mutants have switched to more solvent exposed state than those in ClyAwt^26^. These results indicate that deletion of the β-tongue residues especially may have disrupted the monomeric structure that led to the exposure of the tryptophans to solvent.

### The hemolytic activity of ClyA mutants

**Figure 3:**
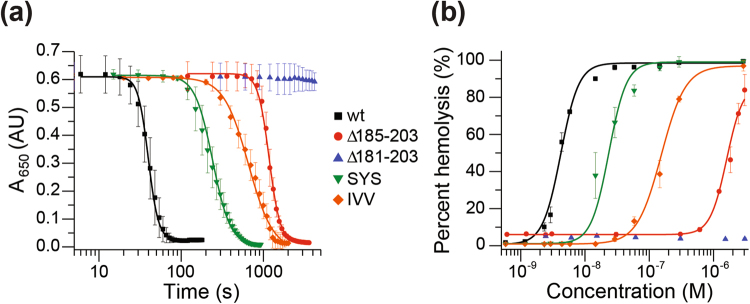
Hemolytic activity of ClyA proteins. (**a**) The time-dependent hemolytic activity of the ClyA proteins was measured as a loss in optical density at 650 nm of the red blood cell (RBC) suspension over time. ClyA proteins (750 µL) were mixed with 250 µL red blood cells to give a final protein concentration of 100 µg/mL and an initial OD_650_ reading of 0.5–0.7. Hemolytic activity for wt (black, 2.90 µM), Δ185–203 (red, 3.05 µM), Δ181–203 (blue, 3.06 µM), SYS (green, 2.91 µM), IVV (orange, 2.91 µM) was measured. (**b**) The concentration-dependent hemolytic activity of ClyA proteins were characterized by measuring A_540_ for the level of hemoglobin released into the supernatant after 15 min incubation of RBCs with varying concentrations of protein. The data points are the average of at least three independent trials and the errors are the standard deviation.

To evaluate the cytolytic activity of the mutants we performed hemolytic assays (Fig. 3). Red blood cells were incubated with ClyA proteins to give an initial OD_650_ reading of 0.5–0.7 and a final protein concentration between 2.9–3.06 × 10^−6^ M. Lysis was monitored at several intervals over time by the decrease in optical density at 22 °C (Fig. 3a). The ClyAwt reached 50% hemolysis after 40.3 ± 0.4s while the mutants were significantly slower. ClyA-SYS mutant reached 50% lysis at 241.7 ± 5.5s, ClyA-IVV at 657.6 ± 15.7s and Δ185–203 at 1174.5 ± 10.5s. Notably, the Δ181–203 mutant did not lyse the cells under these conditions. Next, we monitored cell lysis as a function of toxin concentration (Fig. 3b). Red blood cells were incubated with ClyA proteins ranging from 0.58 nM to 3.06 µM for 15 min at 37 °C. The HC_50_, defined as the concentration of ClyA protein to reach 50% of cell lysis was 4.20 ± 0.23 × 10^−9^ M for ClyAwt. The HC_50_ of the mutants were 2.95 ± 0.3 × 10^−8^ M for ClyA-SYS mutant, 1.54 ± 0.08 × 10^−7^ M for ClyA-IVV and 1.60 ± 0.09 × 10^−6^ M for Δ185–203 respectively, corresponding to a ~10-fold, ~40-fold and ~380-fold reduction in hemolytic activity compared to ClyAwt (Fig. 3b). Under these conditions, the Δ181–203 mutant had no detectable lytic activity. In summary, all of the β-tongue mutants showed a strong reduction in hemolytic activity compared to ClyAwt, while the Δ181–203 mutant had a complete loss of hemolytic ability.

### Membrane-association of β-tongue mutants

It was suggested that the role of β-tongue is to initiate membrane insertion which leads to the assembly of the ClyA monomers at the membrane surface^24^. Because the hemolytic activity of the β-tongue mutants were significantly impaired, we investigated whether the loss in hemolytic activity was due to an inability to associate with membranes.

**Figure 4:**
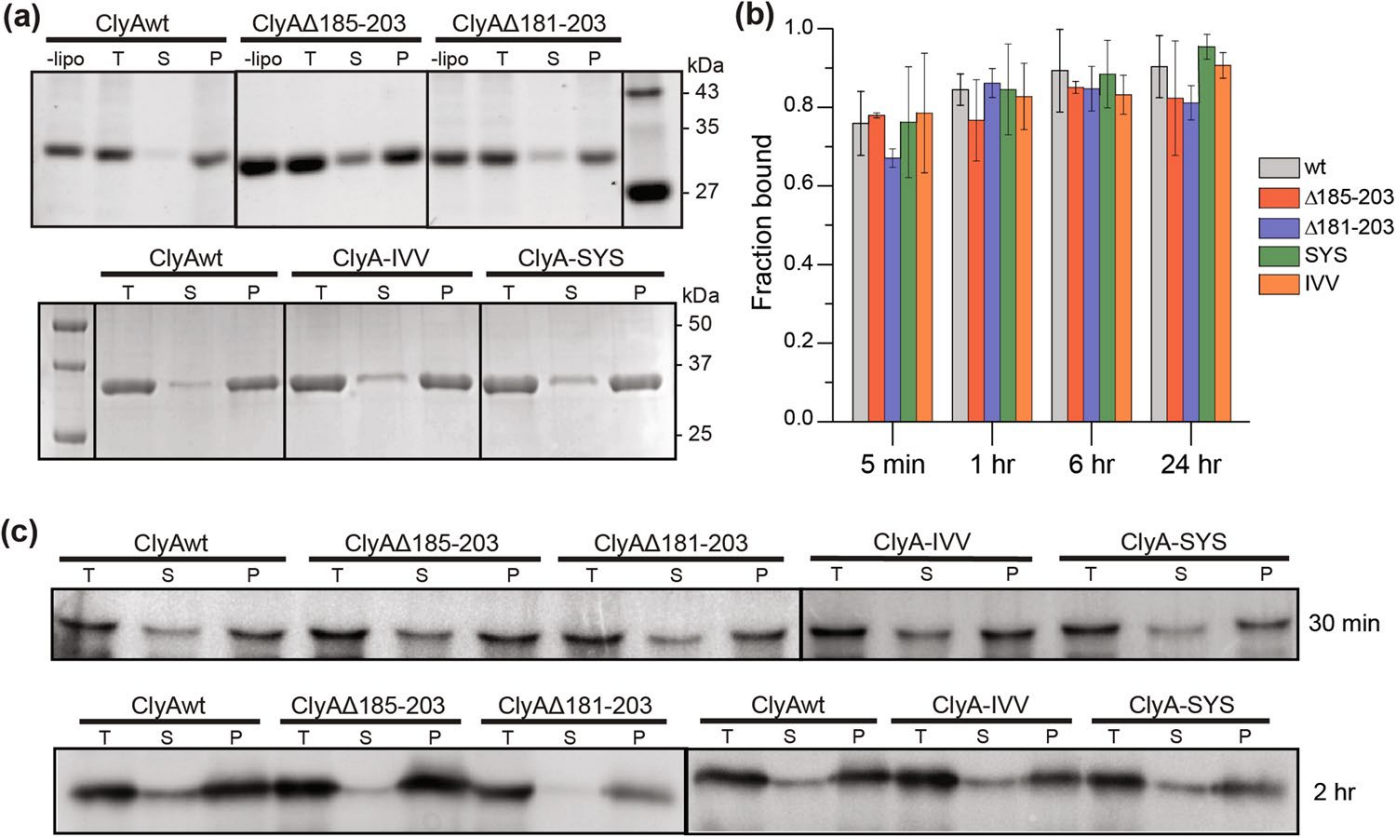
Membrane association assay of ClyA proteins. (**a**) Representative gels after 24 hours incubation of ClyA samples with brain lipid membranes. The no lipid (−lipo) and total samples (T) were not centrifuged. The supernatants (S) and liposome pellets (P) of the liposome-treated samples were collected after separation by ultra-centrifugation. ClyA proteins in all fractions were analyzed with SDS-PAGE. (**b**) Quantitative analysis of proteins found in the pellet (P) fraction compared to that in the total (T) at various incubation times. Percentage of protein bound to the membrane pellets were calculated using ImageJ analysis for the different time points. Fraction bound is the average and the error bar is the standard deviation from three independent trials. (**c**) Western blot analysis of ClyA proteins incubated with red blood cells for 30 mins and 2 hrs. The gels were cropped for conciseness.

We used a membrane pull down assay to assess membrane association ability of ClyA variants to brain lipid vesicles or sheep’s red blood cells. All proteins were incubated with brain lipid vesicles at 23 °C and red blood cells at 37 °C for the indicated times to induce protein-membrane association. The protein-membrane or protein-red blood cell mixtures were then centrifuged and the supernatant and pellet were analyzed by SDS-PAGE and western blot. The fraction of proteins bound to membranes was calculated from the band intensity of pellets compared with the total amount of protein before centrifugation (Fig. 4). After a 5 minute incubation, the fraction of ClyA proteins found in brain lipid membrane was 76 ± 8% for wild type, 78 ± 1% for Δ185–203, 67 ± 2% for Δ181–203, 76 ± 14% for ClyA-SYS and 78 ± 15% for ClyA-IVV (Fig. 4a,b). This indicates that all ClyA proteins associated with membranes with high efficiency, with the Δ181–203 mutant exhibiting a slightly reduced membrane association after 5 min compared to ClyAwt. After one hour incubation, the fraction bound to the pellet reached saturation for all ClyA proteins. Similarly, when the ClyA proteins were incubated with red blood cells, they were mainly associated with the cell pellet (Fig. 4c). After two hours incubation of protein with red blood cells, ~97% of ClyAwt was found in the pellet, while ~95% of Δ185–203, ~73% of Δ181–203, ~81% ClyA-IVV and ~79% ClyA-SYS were in the pellet fraction. These results show that the β-tongue mutations can still bind to vesicle membranes and red blood cell membranes albeit with slightly decreased efficiency.

**Figure 5:**
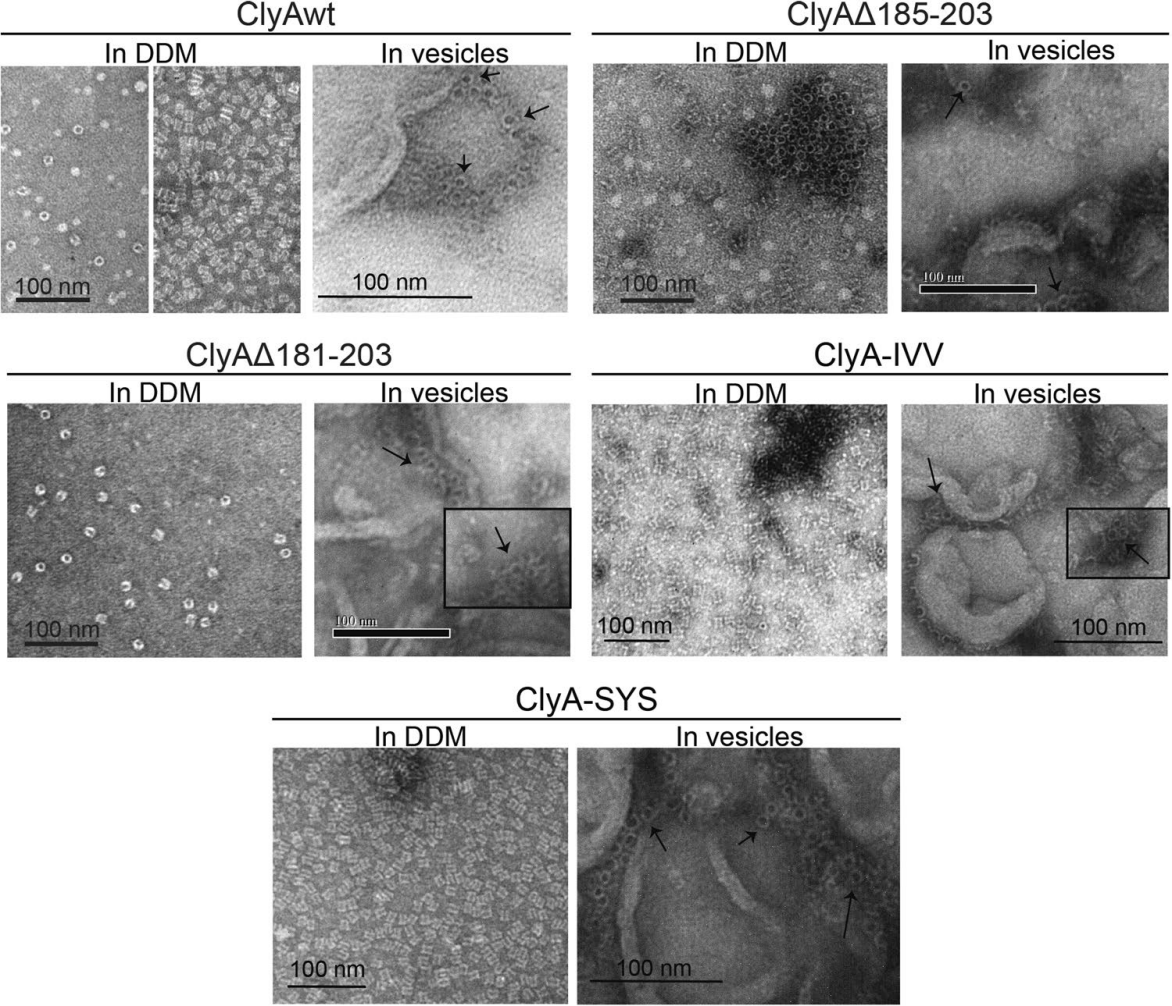
Electron microscopy of DDM-induced and vesicle associated ClyA oligomeric proteins. The images show negatively stained ClyAwt, Δ185–203, Δ181–203, ClyA-IVV and ClyA-SYS proteins that were incubated in 0.1% DDM or in vesicles from brain lipid extract. The two panels of ClyAwt in DDM were images of two batches of independently prepared proteins which exhibited mainly the ring top view (left) and the barrel side view (right) of ClyA. Scale bars represent 100 nm. Black arrows indicate oligomeric rings.

Since the β-tongue mutants were still able to associate with membrane in high efficiency, altered membrane association alone would not explain the severely impaired hemolytic activity of all mutants. We wondered if the alteration of the β-tongue might prevent monomers from forming the oligomeric ring of the pore or pre-pore. Previous studies have used electron microscopy to analyze the structure of ClyA pores that were induced to oligomerize by the addition of detergent dodecyl-D-maltoside (DDM)^20,25^ or octyl-glucoside^27^. We therefore obtained images of DDM-treated proteins as well as proteins that were embedded in vesicle membranes by EM. We found that all five ClyA proteins generated barrel-shaped structures in random orientations on EM grids (Fig. 5). We also tested the heat stability of the DDM-treated proteins and found that the deletion mutants were more heat resistant than ClyAwt, ClyA-SYS and ClyA-IVV (Supplementary Figure S4). Therefore, the loss in hemolytic activity is not caused by the inability to form oligomers or the loss in oligomer stability once formed.

### Single channel recording of ClyA pores

**Figure 6:**
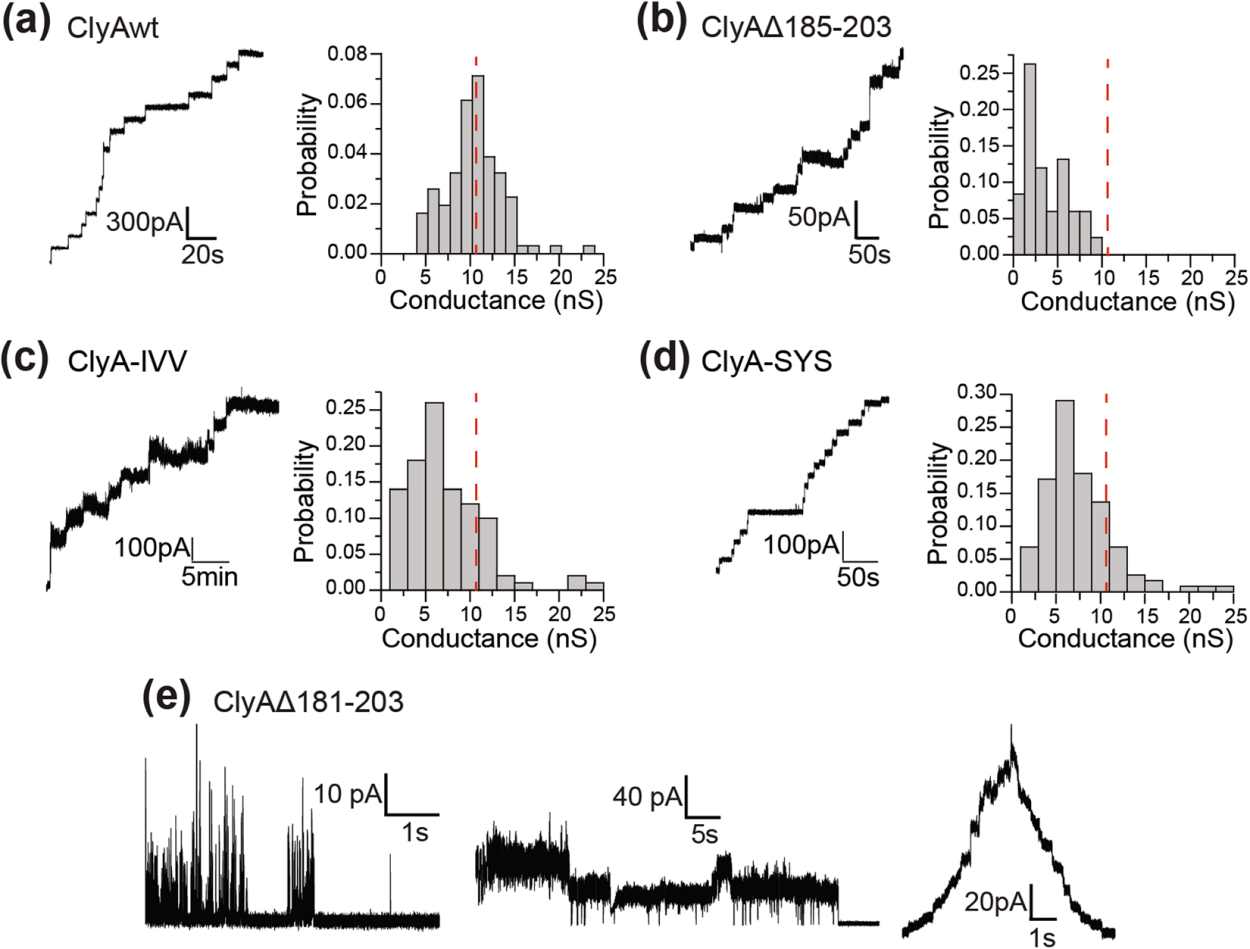
Current recording characteristics of the ClyA proteins. The insertion of consecutive pores of (**a**) ClyAwt and (**b**) Δ185–203 and the population distribution of their conductance. The conductance distributions for (**c**) ClyA-IVV and (**d**) ClyA-SYS. (**e**) The different classes of membrane activity displayed by Δ181–203 mutant. Traces were recorded in 1.0 M KCl, 20 mM Tris-HCl pH 8.0 buffer at +10 mV at a sampling rate of 100 µs using a low pass Bessel filter of 2 kHz. Conductance measurements from at least 100 pore insertions were analyzed. For Δ181–203 over 20 trials were performed.

Next, we investigated the ionic current characteristic of the pores formed by the ClyA proteins by using electrical recording of pores embedded in planar lipid membranes. The ionic conductance of the transmembrane pores is related to their pore dimensions. ClyA proteins were added to the recording chamber and the conductance of pores was calculated from the step-wise increase of ionic current caused by the insertion of individual protein pores (Fig. 6). ClyAwt, Δ185–203, ClyA-IVV and ClyA-SYS produced stable open pores. ClyAwt pores have an average conductance of 10.4 ± 0.04 nS. Δ185–203 pores showed two populations with conductance of 1.9 ± 0.6 nS and 5.5 ± 0.3 nS. The average conductance for ClyA-IVV and ClyA-SYS was 6.4 ± 0.2 nS and 6.8 ± 0.1 nS respectively. Compared to ClyAwt there was a significant reduction in the average conductance for these mutants (Fig. 6a–d). In addition, the Δ181–203 mutant exhibited heterogeneous currents in the planar lipid bilayer which we categorized into two groups: (1) transient current spikes and (2) open pore currents that lasted from seconds to minutes (Fig. 6e). We also observed traces generated by Δ181–203 showing two phases: the early phase showed step-wise increase of the current corresponding to pore insertions, while the later phase showed step-wise decreases in current suggesting pore closures. Our observations suggest that Δ181–203 could not form stable open pores in planar lipid membranes, which could account for its complete loss in hemolytic activity. We surmise that the β-tongue serves as a vital membrane anchor, without which pores can detach from the membrane manifested as stepwise loss in ionic current. Alternatively, the β-tongue pins segments of the helical barrel to the membrane core to hold it open. Without it, the pore can collapse and completely close and possibly re-open transiently. Since the membrane association the Δ181–203 mutant was similar to the ClyAwt pore (Fig. 4), we propose that the β-tongue is more likely to play a role in holding the pore open.

## Discussion

Partial deletion of the β-tongue region resulted in greatly reduced hemolytic activity while complete removal abolished lysis entirely. This observation is consistent with previous studies that showed mutations at or the deletion of the β-tongue caused the loss of hemolytic activity^11,21–23^. However, our data have shown that the β-tongue is not essential for membrane interaction as the full deletion mutant was still able to associate with membranes, albeit with slightly decreased efficiency. Furthermore, the mutation of the β-tongue did not abolish the oligomerization of ClyA, as demonstrated by the EM study of the mutant oligomers. Current recording experiments of ClyA pores further confirmed that the β-tongue deletion mutants were able to form pores on planar lipid bilayers, suggesting that the amphiphilic N-terminal helix (αA) alone is sufficient to mediate membrane association and transmembrane domain formation.

Our single channel electrical current recordings further revealed that the β-tongue partial deletion mutant Δ185–203 and substitution mutants ClyA-IVV and CyA-SYS formed pores of smaller conductance than the wild type. More importantly, the β-tongue full deletion mutant Δ181–203 pore exhibited a short-life span lasting only seconds to a minute, which suggests that the α-helical transmembrane barrel may have switched to a closed conformation. In contrast, a previous electrophysiological characterization of a β-tongue mutant Δ181–203 showed the mutant formed stable pores of broad conductance, half of which were similar to wild type proteins^21^. This observation is contradictory to the results of our hemolytic assays and their own assays in which the complete loss of hemolytic activity was observed for mutant Δ181–203^21^. The contradiction in the previous work was attributed to the mutant’s deficiency in membrane association, which was estimated to be 25% of the ClyAwt level based on the pore insertion rate on DPhPC planar bilayers^21^. If this is the case, then we would expect that a Δ181–203 mutant with only a 4-fold reduction in membrane association should still result in a fairly active toxin, especially if half of the population formed pores similar to the wild type. So, we directly examined membrane retention of ClyA proteins on brain lipid membranes and on red blood cells and found only a slight decrease in association efficiency with our β-tongue deletion mutants. We believe that the slightly reduced membrane association of the β-tongue Δ181–203 alone should not account for the complete loss of its functionality. Although it is logical to think the decreased hemolytic activity of the deletion mutants may be the result of impaired oligomerization efficiency caused by mutations, we observed that the substitution mutants (IVV and SYS) also showed diminished activity, despite none of the residues in substitution mutants are involved in protomer-protomer interactions which stabilize oligomer formation. In addition, even if Δ181–203 mutant had a slower oligomerization efficiency, one might expect it still contains partial hemolytic activity. Instead, we observed a completely inactive toxin after it was exposed to red blood cells for more than one-hour.

**Figure 7:**
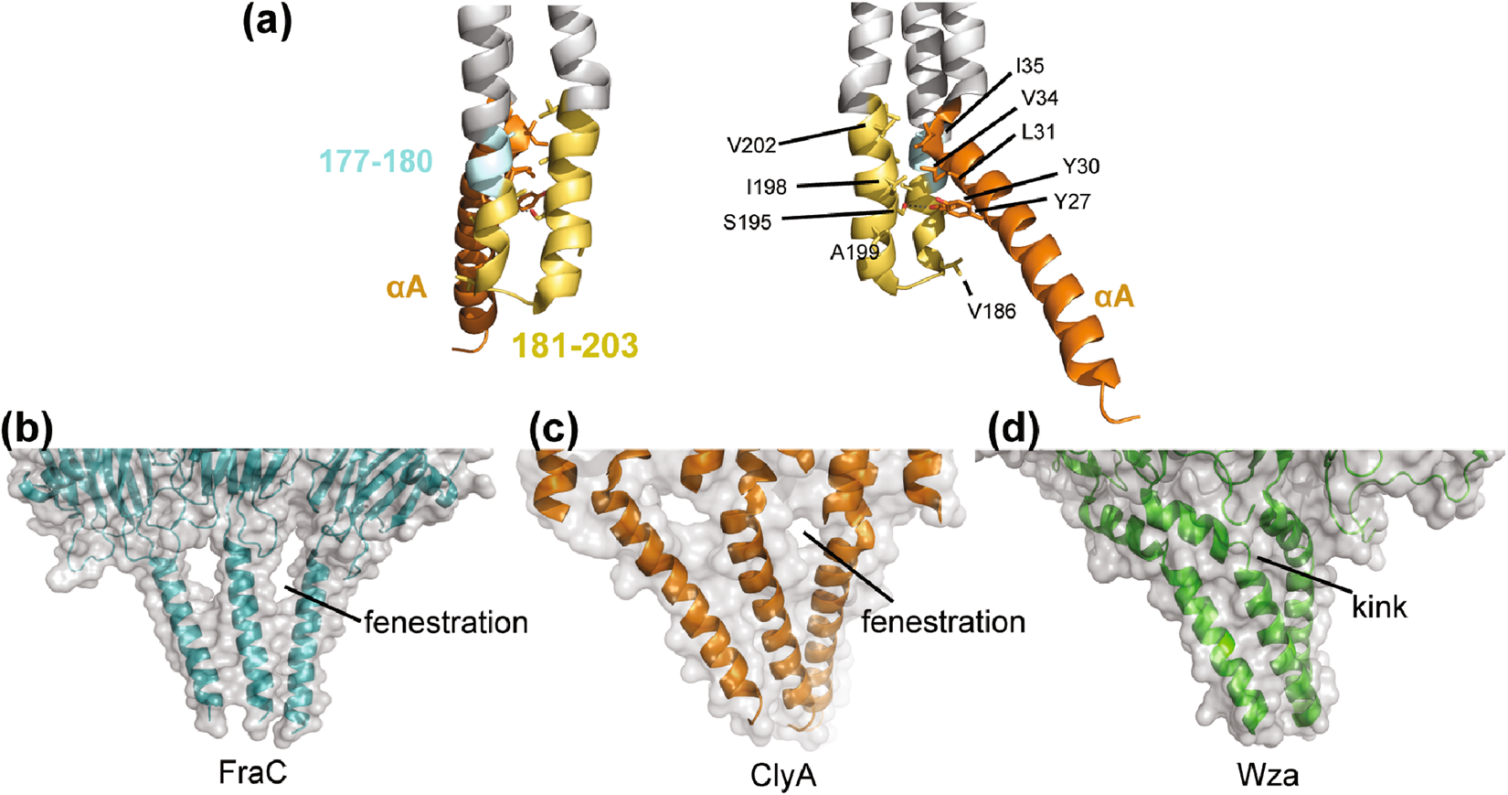
Structures of the α-helical transmembrane regions of ClyA, FraC and Wza channel proteins. (**a**) Hydrophobic interactions and a single hydrogen bond of β-tongue residues (red) with αA residues (blue). (**b**–**d**) Surface representation of the α-helical transmembrane domains of (**b**) FraC, (**c**) ClyA (**d**) Wza. Residues 177–204 were removed from ClyA structure to expose the surface of the transmembrane barrel.

Our single channel studies suggest that removing key residues of the β-tongue led to a smaller or closed transmembrane domain manifested by reduced conductance in all the β-tongue mutants. The crystal structure of the ClyA transmembrane pore shows that residues 181–203 in the β-tongue associated with the αA helix (Fig. 7a,b). Analysis of non-covalent interactions in the ClyA transmembrane pore by PDBsum reveals that 50 interacting residue pairs exist between the αA helix and its surroundings, 31 of which occur between the αA helix-helix packing of adjacent protomers^28,29^. The rest formed between αA and the β-tongue of the same protomer and a neighboring one. Specifically, residues 181–203 of the β-tongue join residues 177–180 to form a helix-turn-helix that packs tightly with αA. Particularly residues I198 and V202 of the β-tongue form hydrophobic interactions with residues Y27, L31, V34 and I35 of αA helix while S195 forms a hydrogen bond with Y30 (Fig. 7). When the interactions between β-tongue and αA were disrupted in the substitution mutants ClyA-IVV and ClyA-SYS, where the I198/V202 and S195 were replaced with alanine respectively, the conductance of the pores were reduced by 40% regardless if the hydrophobicity of the β-tongue was increased or decreased. Apparently, the significant deletions of the β-tongue resulted in partially or completely closed pores. This could be due to Δ181–203 deletion mutant may also alter the orientation of the α-helix 177–180 and disrupt hydrophobic interaction between A179 with the L31 of the neighboring αA in addition to the loss of I198/V202/S195 interactions (Fig. 7). Because of that, we surmised that in order to gain more contact with each other without the two supporting helices formed by the β-tongue, the αA helical domain of Δ181–203 may twist to a larger degree relative to the bilayer normal, which results in a more closed pore compared to other mutants. This speculation will be further investigated by structural studies and molecular dynamics simulation.

Two other structures that have a barrel structure formed by a single transmembrane α-helix are the fragacea toxin C (FraC) produced by sea anemones and the *E. coli* outer membrane protein Wza, which is responsible for exporting polysaccharide from the bacteria^30,31^. The α-helical barrel of the FraC octamer is tightly held by the protein-lipid interactions. Eight fenestrations appear at the promomer-protomer contact interface that are exposed to the lipid bilayer (Fig. 7b). Of note, 12 smaller fenestrations could also be seen between the protomers of dodecameric ClyA after removing the β-tongue region from the ClyA transmembrane pore structure to expose the α-helical barrel (Fig. 7c). In the FraC α-helix barrel, lipids were found to cover these fenestrations by forming interactions with the α-helical peptides. In ClyA, the β-tongue transformed into a two helix bundle that covers up the gap instead. Octameric Wza, exhibits a perfectly sealed barrel wall, yet the transmembrane domain of Wza consists of a 26 amino acid long α-helix with a kink created by a proline at the 11th position. The kink breaks the α-helix into two shorter helices that spans the lipid membrane by 13.8 Å and 26.1 Å, respectively (Fig. 7d). By this arrangement, 42 interacting residue pairs can be found between the two neighboring transmembrane helices within Wza, compared to 22 pairs within FraC and 31 pairs within ClyA as revealed by PDBsum analysis^28,29^. The significantly reduced helix packing in FraC and ClyA likely account for the fenestrations that appear in the barrel. This suggests that a single continuous α-helix with a length >35 Å, which is required to span the lipid bilayer, is unable to pack seamlessly to form a barrel. Unlike FraC, the ClyA β-tongue mutants may not be able to maintain its original helix-helix packing angle because it would expose its negatively charged residues around the fenestrations to the hydrophobic lipid environment (Supplementary Figure S5).

The monomer structures of two α-PFTs, Hbl-B and NheA, exhibit remarkably high structural similarity to the ClyA monomer despite their lack of sequence homology^32,33^. These two *Bacillus cereus* toxins also contain a hydrophobic β-tongue like region that was proposed to form the transmembrane domain. Furthermore, the recently solved crystal structure of Cry6Aa, a Cry insecticidal protein from *Bacillus thuringiensis*, also resembles the structure of the ClyA monomer except that a long hydrophobic loop was found at the corresponding β-tongue region^34^. Future biochemical and structural studies of the transmembrane pores of these ClyA-type αPFTs could further reveal if a common structural arrangement is shared among them during membrane association and penetration.

In summary, the β-tongue mutations reduced or abolished cell lytic activity in the ClyA toxin, seemingly because the mutant may be unable to form a fully open transmembrane domain in the lipid bilayer. Our data suggest the essential role of two helix bundles transformed from the β-tongue region in stabilizing the αA helix barrel structure of the transmembrane domain. Our finding may also provide useful guidance for the design of new α-helical barrel protein pores.

## Methods

### Construction of ClyA β-tongue mutants

The β-tongue deletion ClyA mutants (Δ185–203 and Δ181–203) were obtained by overlap PCR as previously described^35^. PCR was performed using a Phusion polymerase kit (New England Biolabs) with pT7-ClyAwt-His_6_ as a template. The primers for Δ185–203 were 5′-GGTGGTCCAGAAGGAAAACTGATTCCAGAATTG-3′ (forward) and 5′-TCCTTCTGGACCACCGGCTGCGGCACCGGCATATG-3′ (reverse) while those for Δ181–203 were 5′-GCCGGTGGTCCAGAAGGAAAACTGATTCCAG-3′ (forward) and 5′-CTTCTGGACCACCGGCATATGCTTCCTTCCTGATTTTATC-3′ (reverse). ClyA-IVV primers were 5′-GCGGCTGCGGGCGCCGCGGAAGGAAAACTGATTCCAG-3′ (forward) and 5′-GGCGCCCGCAGCCGCAGAATAGGAAATGATTAATC-3′ (reverse) while ClyA-SYS primers were 5′-ATTGCGGCAGCAATTGCTGCGGGCGTAG-3′ (forward) and 5′-AATTGCTGCCGCAATGATTAATCCAAATG-3′ (reverse). The four different PCR products were digested with DpnI at 37 °C for 3 hours to remove the template plasmids. Chemically competent *E. coli* Novablue cells were then transformed with the DpnI mixture. Plasmids from transformed Novablue colonies were digested with NdeI to identify those containing the ClyA genes and then the mutations were confirmed by DNA sequencing.

### Protein expression and purification

The plasmids were transformed into *E. coli* BL21 (DE3). Cells from a pre-culture inoculated from a single colony were grown in 250–500 mL Luria Broth (LB) media at 37 °C to an OD_600_ of 0.6. Isopropyl β-D-1-thiogalactopyranoside was then added to the cell culture at a final concentration of 0.5 mM to induce protein expression. The cultures were grown for 16–18 h at 15 °C and then harvested by centrifugation. The cell pellets were frozen at −20 °C until ready to use. The C-terminal hexa-His-tagged ClyA proteins were purified by Ni-NTA affinity chromatography. Cell pellets were resuspended in 20 mL lysis buffer (50 mM Tris-HCl, pH 8.0, 1 mM EDTA, 0.1 mM phenylmethanesulfonylfluoride). The cell suspensions were then sonicated for 15 min and the lysate was centrifuged at 10,000 × g for 15 min to pellet the cell debris. After passing through a 0.45 µm filter, the supernatant was loaded onto gravity Ni-NTA affinity columns that were equilibrated with cold buffer A (50 mM Tris-HCl, pH 8.0, 150 mM NaCl). His-tagged ClyA proteins were eluted in eluting buffer (50 mM Tris-HCl, pH 8.0, 150 mM NaCl, 150 mM imidazole) after an initial treatment with wash buffer (50 mM Tris-HCl, pH 8.0, 150 mM NaCl, 50 mM imidazole). The eluted proteins were then dialyzed twice at 4 °C for at least 8 h in dialysis buffer (50 mM Tris-HCl, pH 8.0, 5 mM EDTA, 150 mM NaCl). The dialyzed protein was concentrated using 10 kDa cutoff Centricon and further purified using HW-55S gel filtration column (TOSOH) that was equilibrated in 10 mM sodium phosphate buffer pH 7.0, 150 mM NaCl. The ClyA proteins were collected and assayed the same day or stored at −80 °C until use. The purity of the protein (>95%) was verified by sodium dodecyl sulfate-polyacrylamide gel electrophoresis (SDS-PAGE) and visualized using a stain free imaging method (Biorad). Protein concentration was determined by bicinchoninic acid (BCA) assay.

### Gel Filtration of ClyA proteins

ClyA proteins were prepared and analyzed similar to previously described methods^35^. Briefly, purified proteins (100–200 µg) were analyzed by using an analytical Superdex 200 10/300 gel filtration column (GE healthcare) equilibrated in 150 mM NaCl, 20 mM sodium phosphate pH 7 buffer.

### Liquid Hemolysis assay to test pore-forming activity

Sheep’s defibrinated blood (Lampire Biological Laboratories) was rinsed in 50 mL of isotonic buffer (150 mM NaCl 10 mM sodium phosphate pH 7.0) and pelleted at 3,200 × g until the supernatant was colorless/light pink. The red blood cell pellet was then resuspended in isotonic buffer to the indicated diluted percent. For the concentration dependent hemolysis assay, 250 µL of 25% red blood cell suspension were mixed with 100 µL of prepared ClyA proteins of various concentrations ranging from 0.58 nM to 3.06 µM at 37 °C for 15 minutes. Samples were then centrifuged at 20,000 × g for 2 min to pellet any intact red blood cells. The absorbance of the supernatant at 540 nm was measured to determine the relative amount of released hemoglobin protein^36^. Total hemolysis was defined by the incubation of red blood cells in MilliQ water. For the time-dependent hemolysis assay^25^, a total of 100 µg of protein was mixed with isotonic buffer in a disposable cuvette to a final volume of 750 µL. Then 250 µL of 1.56% red blood cell suspension was added to bring protein to a final concentration of 100 µg/mL or between 2.9–3.06 µM. The cuvette was inverted three times and the OD_650_ was measured at 22 °C every 2–20 s. The hemolysis results were plot and fit using Origin 2017.

### Circular Dichroism measurement

ClyA protein samples (0.2 mg/mL; 5.8–6.1 µM) in 10 mM sodium phosphate buffer pH 7.0, 150 mM NaCl were filtered using 0.22 µm filter into 1 mm path-length cuvettes. The CD spectrum was recorded at 0.5 nm/s scanning speed on a Jasco 1500–150 CD Spectrometer from 200–250 nm at 25 °C. The spectra are shown as an average of three scans. For heat stability test of ClyA oligomers, the ClyA samples were first pre-incubated in 0.1% DDM at 23 °C for 30 mins. Half of the mixture was heated at 90 °C for 20 min and then cooled to 25 °C and measured after 10 min equilibration at 25 °C.

### Intrinsic Fluorescence measurement

ClyA proteins were analyzed as previously described^35^. Briefly, 1 mL of 0.88–0.91 µM of ClyA proteins were excited at a wavelength of 280 nm and their emission spectra were collected from 293–407 nm using a PTI fluorimeter at 22–23 °C. The spectrum of the blank buffer (150 mM NaCl, 10 mM sodium phosphate pH 7.0) was subtracted from the spectra of ClyA samples.

### Membrane Preparation

To prepare lipid vesicles, porcine brain lipid extract in chloroform (Avanti Polar Lipids) was dried under nitrogen and then reconstituted in 150 mM NaCl, 10 mM sodium phosphate pH 7.0 buffer at a concentration of 25 mg/mL. After five cycles of freeze-thawing, the lipids were then extruded 31 times through a 0.1 µm filter to generate unilamellar vesicles. For membrane pull down assay, 25 µl of 25 mg/mL lipids was reconstituted in 300 µl centrifugation buffer (150 mM NaCl, 10 mM sodium phosphate pH 7.0, 5% sucrose). The reconstituted lipids were flash frozen and thawed three times and bath sonicated for 60 min at room temperature to prepare membrane. The prepared membranes were then used immediately or stored at 4 °C for up to two weeks.

### Electron Microscopy of ClyA pores

ClyA proteins (0.2–0.5 mg/mL) were either incubated in 0.1% DDM for 16 hours at 4 °C or incubated in vesicles made in a 1:100 protein-lipid mass ratio for 16 hours at 22–23 °C. ClyA proteins incubated in DDM were then diluted 5 times with 0.1% DDM solution while protein in vesicles were undiluted. Carbon coated Parlodion grids were glow discharged for 30s. A volume of 5 µL sample was added to the grid and allowed to rest on the carbon side of the grid for 30s. The excess liquid was removed with a filter paper and the adsorbed protein was stained with 2% uranyl acetate onto the surface. The excess liquid was removed with filter paper and the grid allowed to air-dry for 5 min. The samples were imaged using a FEI Tecnai 12 Spirit electron microscope at 92 or 105 kV.

### Membrane pull-down assay

ClyA proteins at a concentration of 250–300 µg/mL were filtered using a 0.22 µm filter and then centrifuged at 20,000 × g for 10 min to pellet any insoluble aggregates. The proteins in the supernatants were then mixed with vesicle membranes at a ratio of ~1:50 protein:lipid mass ratio. The protein-liposome mixture was incubated for various time periods: 5 min, 1 hr, 6 hr, and 24 hr at 22–23 °C. Thereafter, 20 µl of the protein-lipid mixture was reserved as a sample to indicate the total amount of protein while the rest was ultra-centrifuged at 200,000 × g for 30 min using Optima TLX ultracentrifuge (Beckman Coulter). The supernatant was separated from the membrane pellet while the pellet was resuspended in buffer at the same initial volume prior to centrifugation. The total supernatant and pellet samples were mixed with SDS-Laemmli buffer and analyzed via SDS-PAGE. The percent protein found in the pellet was calculated from the protein band intensity as analyzed by ImageJ.

For the membrane binding assay with red blood cells, 150 ng ClyA proteins were mixed with 100 µl of red blood cells (blood was used within six weeks from the bleed date). The protein blood cell mixture was incubated in a 37 °C water bath for 30 min or 2 hr. Thereafter, 30 µL of the protein blood cell mixture was saved as the sample to indicate the total amount of ClyA proteins. The remaining mixture was centrifuged at 200,000 × g for 30 mins at 25 °C. The total, supernatant and cell pellet samples were run on an SDS-PAGE. The proteins were then transferred onto PVDF membrane (GE Healthcare Life Sciences) in cold transfer buffer (192 mM Glycine, 25 mM Tris, 20% methanol) at constant 350 mA for 100 mins. The membranes were then incubated with gentle shaking in 5% non-fat milk dissolved in TBST buffer (150 mM NaCl, Tris-HCl pH 7.4, 0.1% Tween-20) for 1 hr. After three times washing in TBST, the membranes were then incubated with a 1:10,000 dilution of primary mouse antibody against His_6_ tag (His.H8 clone, Invitrogen) in TBST containing 1% BSA for 16 hrs at 4 °C. Then the membranes were washed three times in TBST and incubated with the donkey anti-mouse HRP secondary antibody (1:20,000 dilution) in TBST containing 1% BSA for 1.5 hrs at 23 °C. The membranes were washed three times in TBST and the fourth wash was in TBS. The membranes were developed using the ECL prime kit (GE Healthcare Life Sciences) and imaged.

### Electrophysiology characterization of ClyA proteins

Experiments were performed as previously described in a chip partitioned into two chambers separated by a 25 µm-thick Teflon film containing a 100-µm diameter aperture^35^. The ClyA proteins were incubated in 0.1% DDM overnight at 4 °C to oligomerize prior to electrophysiology characterization. The Teflon film was treated with a hexadecane/pentane (1:10 v/v) solution and the pentane was allowed to evaporate. Thereafter, 900 µl of 1 M KCl, 25 mM Tris-HCl, pH 8.0 buffer was added to both chambers. 1,2-diphytanoyl-*sn*-glycerol-3- phosphocholine (DPhPC) in pentane (10% w/v) was deposited on the surface of the buffer and the pentane was allowed to evaporate. A Ag/AgCl electrode was placed in each chamber with the *cis* chamber grounded. A bilayer was spontaneously formed by pipetting the solution up and down across the aperture. The proteins (5–10 nM) were added to the *cis* chamber and a voltage of +10 mV was applied across the membrane. The current was amplified with an Axopatch 200B integrating patch clamp amplifier (Axon Instruments, Foster City, CA). Signals were filtered with a Bessel filter at 2 kHz and then acquired by a computer (sampling at 100 µs) after digitization with a Digidata 1320 A/D board (Axon Instruments). Conductance measurements from at least 100 pore insertions unless otherwise stated were analyzed with Clampex 10.7 software.

### Data Availability Statement

All data generated or analysed during this study are included in this published article (and its supplementary information files) and are available from the corresponding author on reasonable request.

## Electronic supplementary material

Supplementary Information
