# Supplementary material for: Disruption of the open conductance in the β-tongue mutants of Cytolysin A
Source: Sci Rep. 2018 Feb 28;8:3796. doi: 10.1038/s41598-018-22009-1 (PMC5830503; doi:10.1038/s41598-018-22009-1)
Supplement: Supplementary file 1 — Supplementary Information [file 41598_2018_22009_MOESM1_ESM.docx]

Disruption of the open conductance in the β-tongue mutants of Cytolysin A

Monifa A. Fahie^1,2^, Lucas Liang^2^, Alzira R Avelino^1^, Bach Pham^2^, Patanachai Limpikirati^2^, Richard W. Vachet^1,2^ and Min Chen^1,2^*

1. Molecular and Cellular Biology Program; 2. Department of Chemistry; University of Massachusetts Amherst, Amherst, MA 01003

**Table of Contents Page #**

Supplementary Methods S2

Supplementary Figure S1 S3

Supplementary Figure S2 S4

Supplementary Figure S3 S5

Supplementary Figure S4 S6

Supplementary Figure S5 S7

Supplementary Table S1 S8

**SUPPLEMENTARY METHODS:**

Mass Spectrometric analysis of ClyA proteins

ClyAwt samples were prepared in 50 mM ammonium acetate (NH_4_Ac) pH 7.0. Mass spectra were acquired on a Bruker MicroTOF-II (Billerica, MA) hybrid quadrupole - time of flight mass spectrometer. The electrospray ionization source was operated using a positive mode at a needle voltage of 2800 V. The capillary temperature was set to 220°C. The mass spectra from three independent trials were analyzed by using Bruker Data Analysis Version 4.0 software. Once the charge state of each peak was assigned, the average molecular mass of protein sample was calculated automatically. The ClyA mutants were desalted in distilled water and prepared at ~15 µM. ClyA mutants mass spectral data were obtained at the University of Massachusetts Mass Spectrometry Center. Mass spectra were acquired on a Bruker MicroTOF-II (Billerica, MA) hybrid quadrupole - time of flight mass spectrometer. The electrospray ionization source was operated using a positive mode at a needle voltage of 3500 V. The capillary temperature was set to 200°C.

Gel permeation chromatography coupled with multi-angle dynamic light scattering (GPC-MALS)

ClyA proteins were first filtered using 0.22 µm filter then centrifuged at 20,000 x g for 20 min and 50 µl of the sample was injected onto a TSKgelG3000SW_XL_ (TOSOH Bioscience) column using an Agilent HPLC. The proteins were eluted in 10 mM sodium phosphate buffer pH 7.0, 150 mM NaCl and their dynamic light scattering profiles measured by an Optilab T-rex and Dawn Heleo II (Wyatt Technologies). The baselines of the chromatograms were corrected and the protein peaks were selected to automatically determine the molar masses of the protein. the weight average molecule weight (M_w_) and the number average molecule weight (M_n_) were obtained from three measurements of two separately purified protein. The polydispersity index was also calculated as a measure of the heterogeneity of the protein sample.


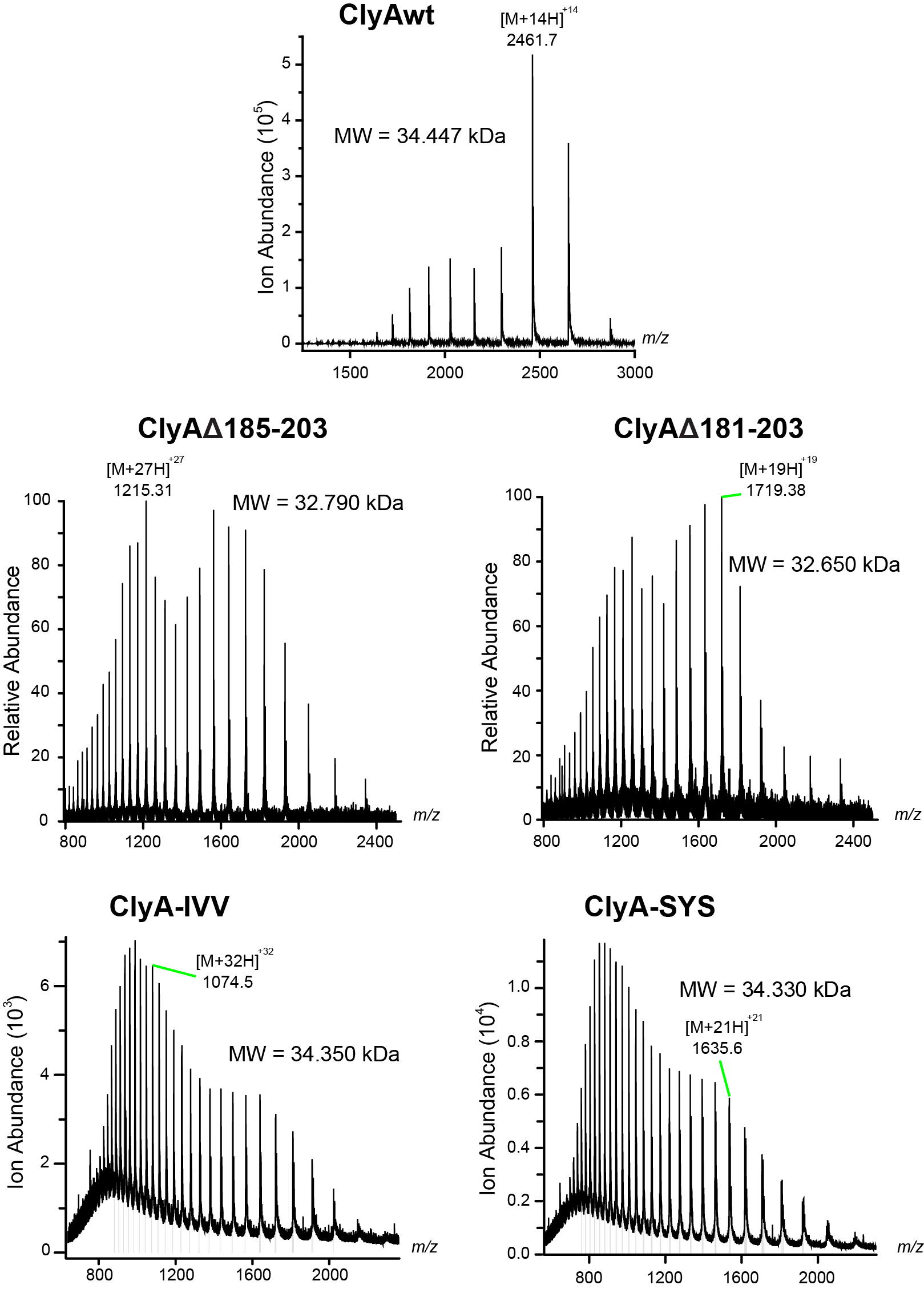


**Supplementary Figure S1:** Mass spectra of ClyAwt and β-tongue mutants. The experimental molecular weight (MW) of WT, Δ185-203, Δ181-203, IVV and SYS are 34.447, 32.790, 32.650, 34.350 and 34.330 kDa respectively while the theoretical MW are 34.450, 32.787 ,32.649, 34.352 and 34.327 kDa respectively. The spectra shown are representative figures from three independent trials. ClyAwt was prepared at ~13 µM in 50 mM NH_4_Ac pH 7.0, then directly injected at a flow rate between 3-4 µL/min. Each spectrum was recorded for at least 5 min and averaged. The ClyA mutants were prepared at ~15 µM and desalted in water. Mass spectral data of ClyA mutants were obtained at the University of Massachusetts Mass Spectrometry Center using a similar method to ClyAwt.


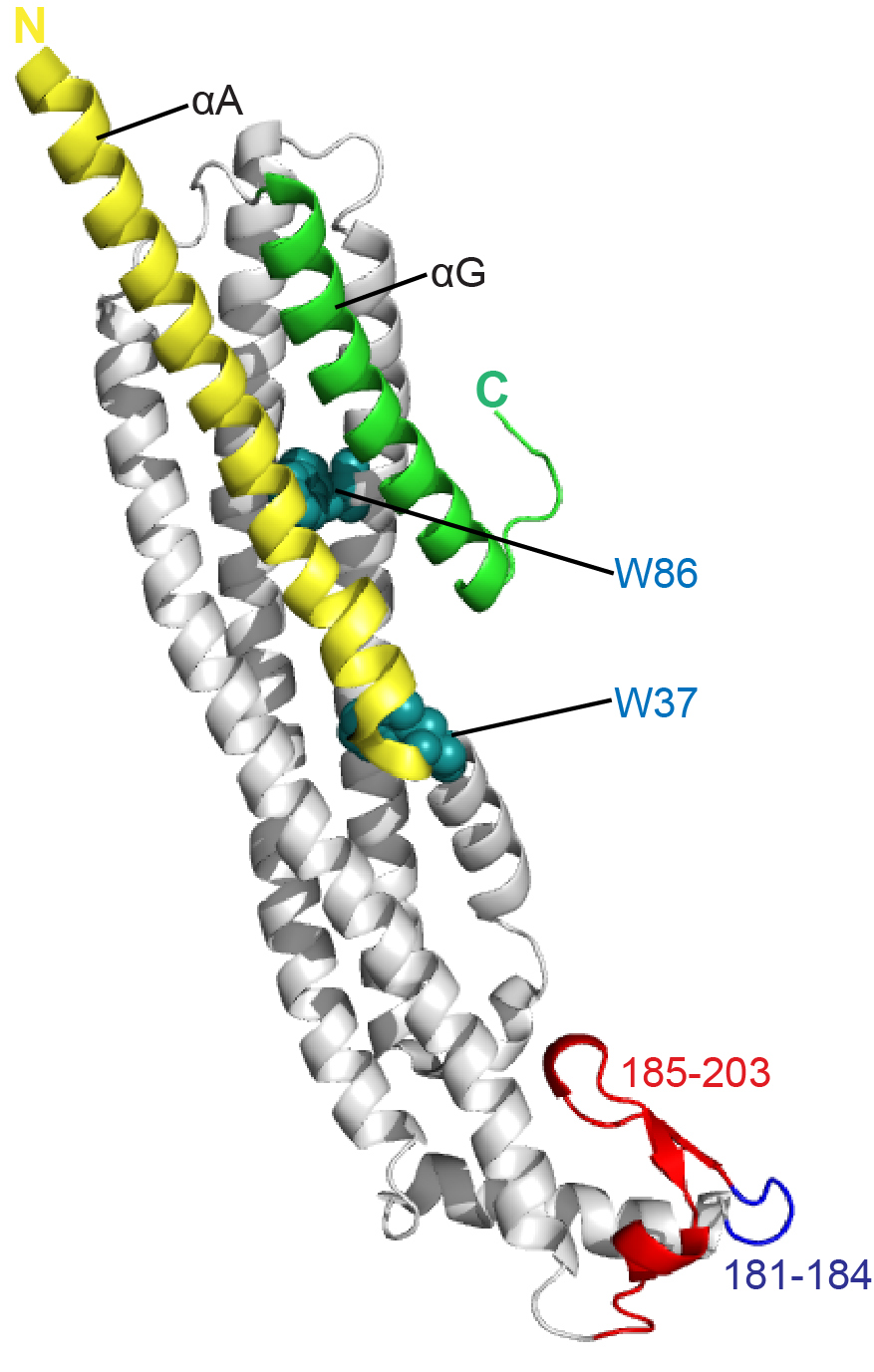


**Supplementary Figure S2:** Schematic of the identity and location of the tryptophan (W) residues in ClyA proteins. The figure depicts the ClyA monomer using PDB 2QOY and shows the tryptophan residues in relation to the β-tongue region (blue and red) and the αA at the N-terminus and the αG at the C-terminus (yellow and green respectively). There are two tryptophan residues W37 and W86 (teal spheres) which are buried in the hydrophobic core of the monomer.


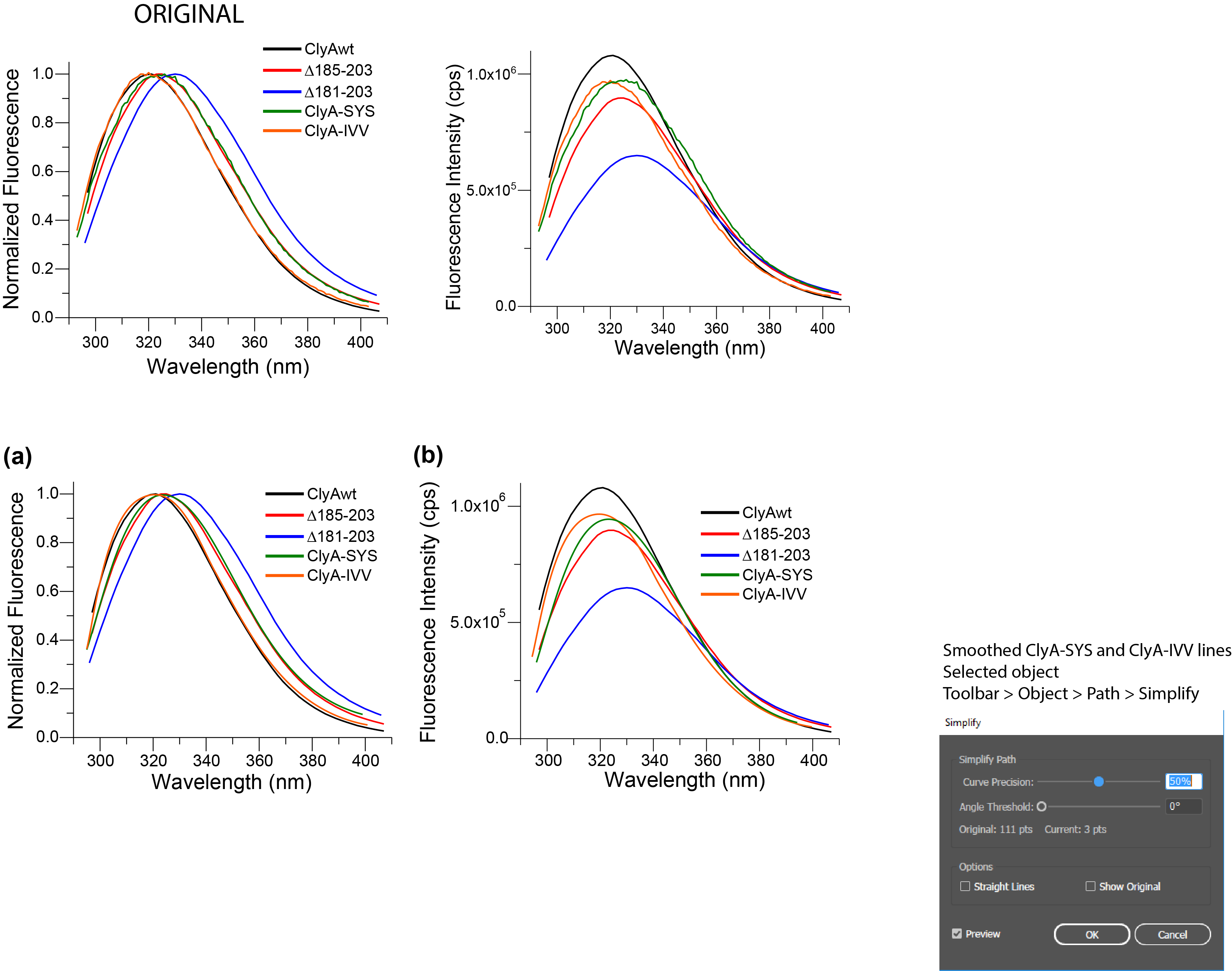


**Supplementary Figure S3:** Intrinsic fluorescence emission spectra of ClyA samples. ClyA proteins were excited at 280 nm and their fluorescence measured from 293-407 nm. Approximately 30 µg/ml (0.88 – 0.91 µM) of protein was prepared in 150 mM NaCl, 20 mM sodium phosphate buffer pH 7.0.

**
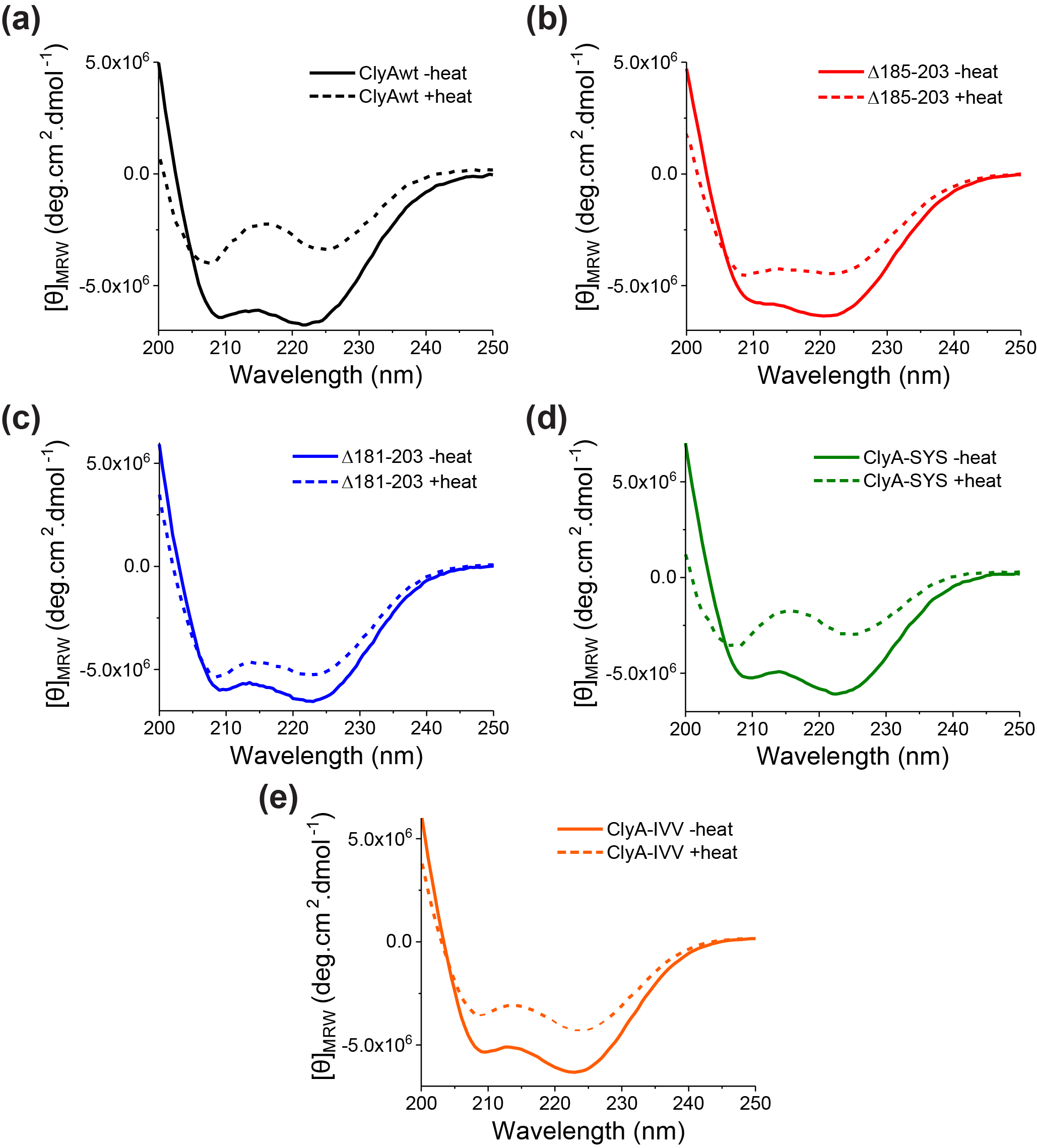
**

**Supplementary Figure S4:** CD melting analysis of ClyA DDM-triggered oligomers with and without heat denaturation/aggregation. The proteins were heated at 90°C for 20 min and then allowed to cool to 25°C before obtaining CD spectra from 200 – 250 nm. All CD spectra were obtained at 25°C for DDM-triggered oligomers of (a) ClyAwt (b) Δ185-203 (c) Δ181-203 (d) ClyA-SYS and (e) ClyA-IVV.


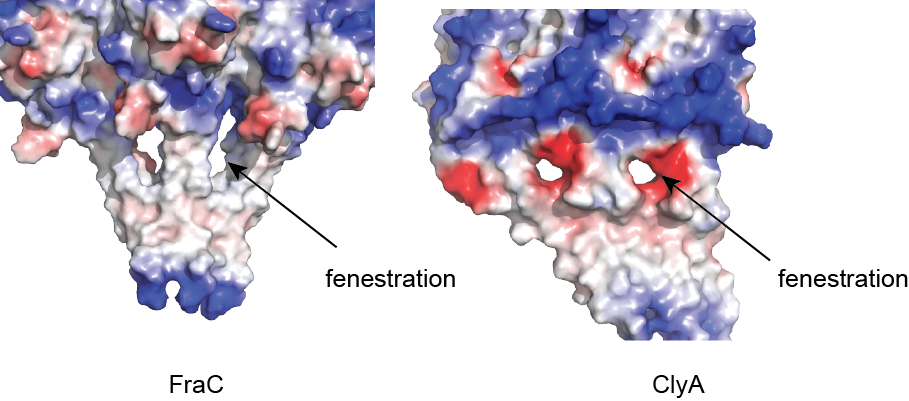


**Supplementary Figure S5:** Comparison of surface electrostatic potentials of the transmembrane domains of FraC and ClyA. In ClyA, the β-tongue regions were manually removed to expose the transmembrane α-helix barrel.

**Supplementary Table S1:** Molecular masses of ClyA proteins determined by GPC-MALS.

| **Molecular Mass (kDa)** | Wt | Δ185-203 | Δ181-203 |
| --- | --- | --- | --- |
| **M_w_** | 34.67 ± 0.81 | 69.15 ± 2.85 | 92.58 ± 4.80 |
| **M_n_** | 34.43 ± 0.84 | 69.04 ± 2.86 | 92.47 ± 4.71 |
| **Polydispersity Index (M_w_/M_n_)** | 1.01 ± 0.04 | 1.0 ± 0.01 | 1.00 ± 0.01 |
